# Meso-Scale Observations of the Evolution of Matrix/Filler Interface Dewetting During NEPE Propellant Aging

**DOI:** 10.3390/polym18111325

**Published:** 2026-05-27

**Authors:** Zebin Chen, Xueren Wang, Zijie Zou, Hongfu Qiang, Mingjian Wang, Yake Wu

**Affiliations:** 1 Qingzhou High-Tech Research Institute, Qingzhou 262500, China; 15229233964@163.com (Z.Z.); victory816@sina.com (M.W.); 19284224246@163.com (Y.W.); 2 Zhijian Laboratory, Rocket Force University of Engineering, Xi’an 710025, China; rocketwangxr@126.com (X.W.); qiang@263.net (H.Q.); 3 School of Astronautics NPU, Northwestern Polytechnic University, Xi’an 710025, China

**Keywords:** solid rocket motor, NEPE propellant, mesoscale, dewetting, matrix/filler interface

## Abstract

To clarify the evolution of dewetting during the aging of NEPE propellant during long-term storage and more intuitively reveal the impact of aging on dewetting behavior, we used micro-CT to scan NEPE propellant samples subjected to 20% constant strain at different points during aging. After image processing, the internal pores of the samples were extracted, porosity was calculated, and the law of the variation in dewetting behavior at the matrix/filler interface during aging was analyzed. Additionally, we used SEM technology to scan the tensile fracture surfaces of the NEPE propellant samples, observing the aging evolution of the matrix and matrix/filler interface on the fracture surfaces. Along with conducting a micro-CT test, we further explored the changes in bonding performance at the matrix/filler interface during aging. The micro-CT scanning results indicated that dewetting was evident in unaged samples under constant-strain loading, resulting in numerous petal-shaped pores with a significant volume. As aging progressed, the number of petal-shaped pores gradually decreased, and porosity dropped significantly. The SEM scanning results show that the matrix gradually softened during aging, encapsulating solid particles more tightly. Based on all the experimental results, debonding in the NEPE propellants became progressively less pronounced with aging, and interfacial adhesion between the matrix and filler improved. These results provide support for enhancing NEPE propellants’ matrix/filler interfacial bonding strength and, consequently, improving their mechanical properties.

## 1. Introduction

NEPE (Nitrate-Ester-Plasticized Polyether) propellant is a polyether propellant plasticized with mixed nitrate esters [1]. It offers advantages such as high energy and excellent low-temperature mechanical properties, making it a focal point in the development and use of high-energy propellants in the 21st century [2]. One of the common ways solid propellants are damaged during long-term storage is aging, leading to a decrease in mechanical, combustion, energy, and safety performance indicators. The decline in mechanical properties is often the most pronounced [3,4]. Aging-induced failure of solid propellants can largely be divided into three types: matrix, filler, and matrix/filler interface failure. Of these three solid-propellant components, the matrix/filler interface is the weakest [5,6]. Under external loads, it is prone to damage, leading to debonding between the matrix and the filler. This debonding-induced failure of the matrix/filler interface is referred to as “dewetting”. Dewetting can create pores within the propellant, which can lead to stress concentration during subsequent loading processes and is a significant factor contributing to the overall degradation of its mechanical properties [7,8]. In this context, an in-depth investigation of the dewetting behavior of NEPE propellant during aging is of significant theoretical importance for and provides guiding engineering insight into performance evaluation, life prediction, and maintenance strategy formulation regarding solid rocket motors.

Domestic and international scholars have conducted extensive research at the micro- and meso-scales on dewetting in solid propellants. Micro-computed tomography (micro-CT) has been utilized to characterize the three-dimensional meso-structures of solid propellants. By scanning specimens under various types of tensile strain, researchers have successfully reconstructed the initiation and propagation of cracks under such strain [9,10]. Additionally, the meso-structural evolution behaviors of hydroxyl-terminated polybutadiene (HTPB) and NEPE propellants have been investigated. Micro-CT has been employed to observe dewetting in propellants during uniaxial tensile testing, and porosity has been used to quantitatively characterize the degree of meso-scale damage [11,12]. Studies have also characterized variations in mean grayscale values and average porosity within propellants during dynamic processes under tensile strain. It has been concluded that porosity can be used to quantitatively represent dewetting evolution, with pore changes having been divided into four distinct stages. In addition, the influence of aging on mesoscopic damage to propellants has been investigated [13,14,15]. Digital image processing has been applied to in situ scanning electron microscopy (SEM) images of propellants under tension. The meso-structural fractal dimension served as a quantitative indicator, thereby enabling a quantitative analysis of the meso-scale dewetting damage process [16,17]. A three-dimensional computational framework has been developed for simulating interfacial debonding evolution in reinforced elastomers subjected to finite deformations. Using an exponential cohesive zone model (CZM), researchers have conducted numerical investigations to examine debonding progression in both single- and four-particle systems [18,19]. The cohesive zone element method has also been employed to construct a finite-element model for studying dewetting damage in propellants. The results indicated that the emergence of “crack pairs” under an external load was the internal cause of dewetting damage [20]. Molecular dynamics (MD) modeling of the HTPB/ammonium perchlorate (AP) interface was carried out, considering AP decomposition during aging. Uniaxial tensile simulations were performed to evaluate the debonding process, and comparisons with experimental results were made. The findings showed that thermal decomposition aging of AP significantly reduced the mechanical performance of the interface [21]. Additional MD simulations explored the effects of different degrees of oxidative crosslinking and chain scission degradation on the performance of the matrix/filler interface. Interface models for AP/HTPB and aluminum/HTPB were constructed, and the simulation results revealed that interfacial binding energy increased with the degree of chain scission degradation while decreasing with the degree of oxidative crosslinking [22]. Models of the matrix/HMX and matrix/RDX interfaces were also constructed, incorporating nitrate ester decomposition and changes in crosslink density during NEPE propellant aging. Separate analyses demonstrated that interfacial adhesive performance improved with nitrate ester decomposition but reached suboptimal levels when the crosslink density was either too high or too low [23].

Currently, research on the meso-scale dewetting behavior of NEPE propellant has been relatively extensive. However, studies focusing on aging-induced dewetting remain limited, and our understanding of how matrix/filler interfacial bonding evolves during aging is still incomplete. Therefore, it is essential to conduct meso-scale characterization experiments specifically targeting the matrix/filler interface, directly observe the evolution of aging-induced dewetting, systematically analyze the evolution behavior of interfacial bonding performance, and, ultimately, gain deeper insights into the underlying mechanisms governing interfacial degradation.

## 2. Materials and Methods

### 2.1. High-Temperature Accelerated Aging Test

The samples used in this paper are all standard dumbbell-shaped test pieces of NEPE propellant provided by the Sixth Academy of China Aerospace Science and Industry Corporation. In this NEPE propellant, PEG is used as the binder; N-100 serves as the curing agent to form the binder system, with NG and BTTN as plasticizers; AP and HMX are used as solid fillers; and Al powder is used as the fuel. Its basic composition is shown in Table 1.

According to GJB 10021-2021 “Accelerated Aging Test Method for Composite Solid Propellants” [24], high-temperature accelerated aging tests were conducted on NEPE propellant samples. The aging temperature was 60 °C, and the tests were carried out for a total of 12 weeks. Sampling points were set at weeks 0, 1, 2, 3, 4, 5, 6, 7, 9, and 12, with four samples taken at each sampling point. The high-temperature aging chamber used was produced by Xiamen Jinheyuan Technology Co., Ltd. (Xiamen, China), with equipment model JHY-H-80L.

### 2.2. Research on Micro-CT Scanning Method for Constant-Strain Tensile Test Specimens

**Figure 1:**
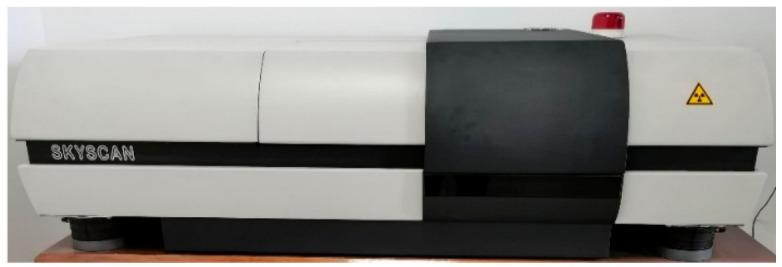
Skyscan 1172 micro-CT.

The Skyscan 1172 micro-CT (Belgian SkyScan Company, Kontich, Belgium) used in this study is shown in Figure 1. The fixed-strain fixture was combined with the sample and fixed to the micro-CT sample table. The scanning parameters are as follows: working voltage, 80 KV; current, 133 μA; CCD camera resolution, 4000 × 2672; spatial resolution, 1.4 μm; sample table height, 48 mm; scanning angle, 360°; and rotation angle increment, 0.3°. The scanned two-dimensional projection images were reconstructed to obtain a set of two-dimensional slice images with the Z-axis as the normal direction. After the slice images were recombined, a three-dimensional result was obtained.

Applying a certain load to NEPE propellant samples facilitates the observation and study of dewetting. During micro-CT scanning, applying 20% constant strain to NEPE propellant results in noticeable dewetting, without causing significant damage to the propellant sample [11].

**Figure 2:**
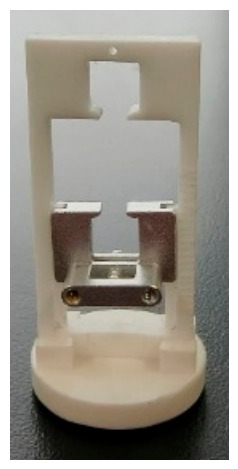
Fixed-strain fixture and its components.

Due to the small size of the micro-CT sample stage and the sensitivity of the scanning process to interference, a specialized fixture for applying fixed strain to the propellant had to be designed. This fixture had to be capable of applying a predetermined strain precisely while avoiding interference with the micro-CT scanning results. The designed fixed-strain fixture is shown in Figure 2. The fixture consists of three main parts: a frame, lower clamp, and base. The fixture frame is made of polytetrafluoroethylene (PTFE), which effectively prevents the generation of artifacts or interference in micro-CT scans. The lower end of the frame is equipped with a slide rail, and the right side of the frame features a scale with 1 mm graduations, allowing for precise vertical sliding movement. The lower clamp is made of 6061 aluminum. The interior of the clamp has a groove. Tightening the special double nut causes the lower end of the groove to contract, thereby clamping the clamp onto the slide rail and achieving fixed displacement.

**Figure 3:**
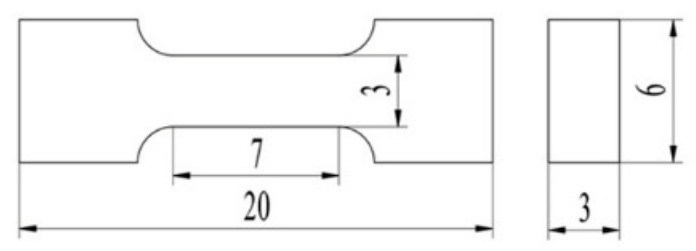
Dimensions of the small dumbbell-shaped specimen.

A specialized cutting tool was used to cut the specimen into a small dumbbell-shaped test piece measuring 7 mm × 3 mm × 3 mm. The specific dimensions are shown in Figure 3. The cutting location was the central gauge section of the standard dumbbell-shaped test piece. The special double nuts of the fixture were loosened. After the specimen was installed, it was stretched downwards by 1.4 mm. The nuts were then tightened to secure the fixture, achieving the effect of applying a fixed 20% strain.

### 2.3. SEM Method for Analyzing the Microscopic Morphology of Tensile Fracture Surfaces

Selecting the tensile fracture surface of the NEPE propellant dumbbell-shaped specimen for observation made dewetting more obvious, aiding in subsequent analysis and characterization. The tensile method referred to is QJ924-85 “Method for Uniaxial Tensile Testing of Composite Solid Propellants” [25].

A Zeiss Sigma300 scanning electron microscope (Carl Zeiss AG, Jena, Germany) was employed. As the solid propellant itself is non-conductive, its surface was sputter-coated with a layer of gold prior to SEM observation to enhance conductivity. The gold-coated sample was placed in the sample chamber, and the chamber was evacuated to maintain a vacuum level below 100 Pa, preventing airborne particles from interfering with the electron beam. The operating voltage and working distance were set to 5 kV and 4.3 mm, respectively, and the InLens detector was selected for signal reception. Observations of the matrix morphology and its adhesive interfaces with AP, HMX, and Al powder were conducted at magnifications of 100× and 2000×.

## 3. Results and Analysis

### 3.1. Analysis of Micro-CT Experimental Results

#### 3.1.1. Micro-CT Scan Image Processing

**Figure 4:**
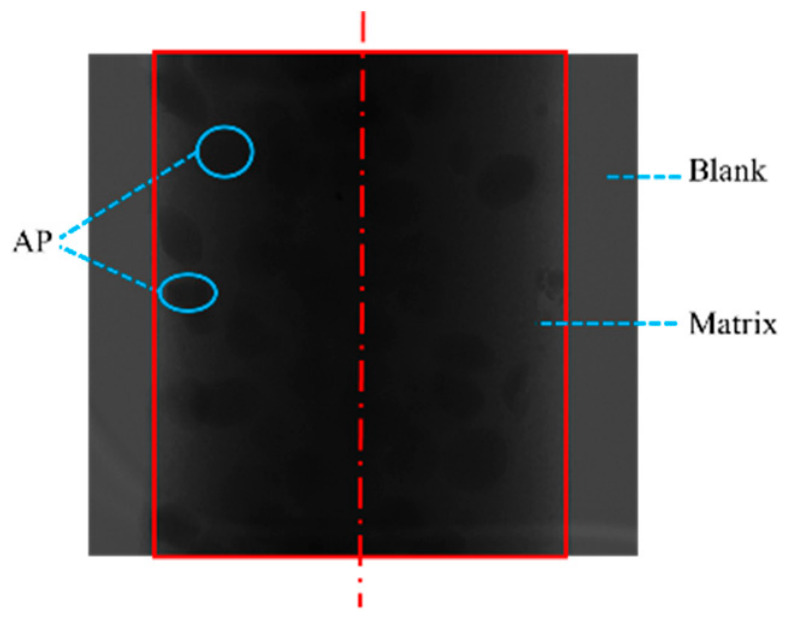
Raw micro-CT scan image.

**Figure 5:**
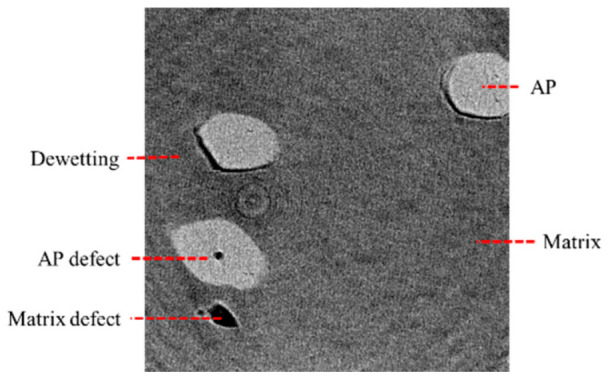
Reconstructed 2D slice image.

A raw micro-CT scan image obtained after applying a fixed 20% strain to the small NEPE propellant specimen is shown in Figure 4. The reconstructed 2D slice image is shown in Figure 5.

Based on the specific composition of the NEPE propellant specimen, the various structural components can be identified from the grayscale distribution and morphological features in the micro-CT scan images. AP exhibits stronger absorption of X-rays, resulting in a darker appearance in the original (raw, unprocessed) image. The binder matrix has a weaker X-ray absorption capacity compared to AP, thus appearing lighter. The reconstructed image displays an inverse grayscale characteristic relative to the original image. Since HMX particles are smaller and their X-ray absorption characteristics are relatively close to those of the matrix, they are difficult to distinguish clearly in the CT scan images.

**Figure 6:**
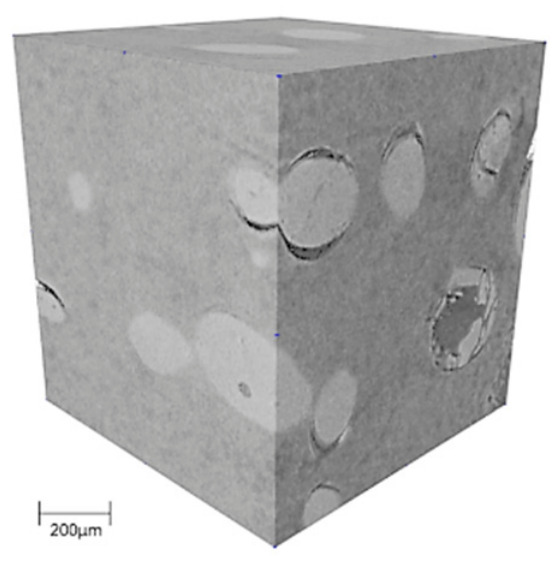
CT image of the unaged specimen under 20% fixed strain.

The reconstructed 2D slices were used for three-dimensional reconstruction via Avizo image-processing software (version Avizo 2020), generating a 3D volumetric structure through stacking. A 3D meso-morphology image of the NEPE propellant specimen is shown in Figure 6. Its grayscale pattern is consistent with that of the 2D slice images.

#### 3.1.2. Aging-Induced Dewetting and Pore Evolution

**Figure 7:**
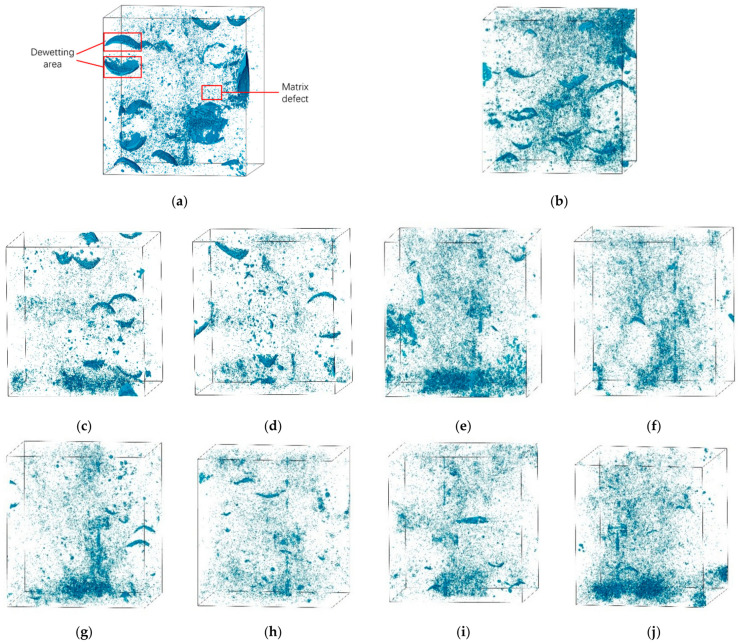
Micro-CT scan results for specimens with aging for different durations: (**a**) unaged; (**b**) aged for 1 week; (**c**) aged for 2 weeks; (**d**) aged for 3 weeks; (**e**) aged for 4 weeks; (**f**) aged for 5 weeks; (**g**) aged for 6 weeks; (**h**) aged for 7 weeks; (**i**) aged for 9 weeks; and (**j**) aged for 12 weeks.

After processing the 3D image with filtering and denoising, the internal pores of the propellant were extracted based on grayscale differences. The threshold segmentation method was applied to the 2D slices to isolate pore defects and dewetting regions, which were then independently reconstructed in 3D. This process yielded 3D images containing only pore defects and dewetting areas. Representative scan results from specimens aged for various amounts of time were selected, and their internal pore images are shown in Figure 7.

Shown after image processing, the internal defects of the solid propellant extracted via micro-CT scanning are displayed as blue areas. The smaller, point-like defects are primarily located within the binder matrix. Due to the relatively loose structure of the polymeric matrix material, numerous micropores and defects were generated under the fixed strain applied. The internal dewetting sites in the solid propellant, as revealed by micro-CT scanning, present as distinct arc-shaped, petal-like structures, characterized by greater volume and prominent morphological features. The two dewetting regions outlined in red in Figure 7a represent upper and lower interface debonding between the same AP particle and the matrix. This figure directly shows that for the unaged NEPE propellant under 20% fixed strain, petal-like dewetting defects are relatively abundant, indicating more severe dewetting. As aging time increases, the petal-like defects gradually decrease in number, and their morphology becomes less distinct. Additionally, the thickness of the petal-like structures also diminishes significantly. When accelerated aging progresses beyond 4 weeks, the petal-like defects are no longer prominent. By 9 to 12 weeks of accelerated aging, the petal-like defects have essentially disappeared and are difficult to identify, indicating a relatively low degree of dewetting at this stage. The experimental results demonstrate that, compared to the unaged specimen, the degree of dewetting in the aged propellant was reduced.

#### 3.1.3. Analysis of the Variation in Porosity with Aging

**Figure 8:**
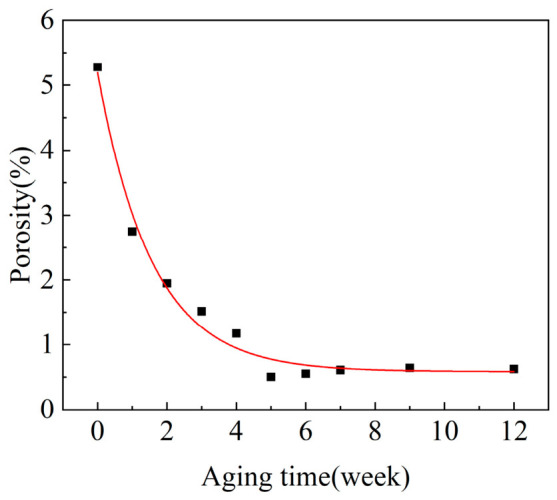
Variation of porosity with aging.

To more clearly and quantitatively characterize the variation in the degree of dewetting with aging time, the defect volume and porosity (the ratio of pore volume to total volume in the micro-CT scan results) of propellant specimens aged for different durations were calculated. A curve illustrating the change in porosity with aging was plotted, as shown in Figure 8.

As shown in Figure 8, under 20% fixed strain, the porosity within the matrix showed a trend of declining with an increase in aging time. During the 0–5-week aging period, the porosity decreased rapidly. From 5 to 12 weeks of aging, the trend leveled off, with no significant changes in porosity. The highest porosity was observed in the unaged state, at 5.28%. In the 5–12-week aging period, the porosity stabilized between 0.5% and 0.65%. In conjunction with Figure 7, it is evident that the petal-like pores that formed during the aging process have a relatively large volume and contribute significantly to the porosity value. In contrast, the point-like pores occupy a very small volume and have a negligible impact on the porosity value. Therefore, the porosity value essentially reflects the changes in the petal-like pores and can be used to indicate the degree of dewetting at the matrix/filler interface of the propellant specimen.

Consequently, based on the experimental results, it can be concluded that during the 0–12-week aging process, the adhesive performance at the NEPE propellant matrix/filler interface improved over time. After short-term aging, the matrix and filler were less prone to dewetting.

### 3.2. Analysis of SEM-Derived Experimental Results

#### 3.2.1. Analysis of SEM Scanning Images

**Figure 9:**
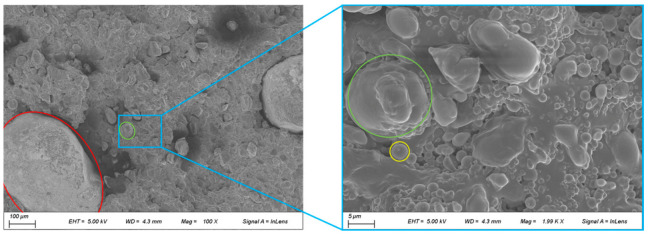
Magnified images (100× and 2000×) of the tensile fracture surface of the unaged specimen.

The fracture surfaces of the specimens after uniaxial tensile testing were scanned using SEM. Images of the unaged specimen’s fracture surface at magnifications of 100× and 2000× are shown in Figure 9. The 2000× image is an enlarged view of a characteristic area selected from the 100× image for detailed observation.

Based on the known composition of the NEPE propellant, the type of solid filler particles can be identified according to their size and shape. In the 100× magnification image, the larger elliptical particles are AP (ammonium perchlorate) particles. The AP particle on the left side (outlined in red) has undergone severe debonding from the matrix, exhibiting a distinct dark dewetting area under SEM. Its shape is petal-like, consistent with the dewetting area observed in the micro-CT scanning results. A similar interfacial debonding phenomenon is observable in another AP particle on the right side of the image. The smaller particles outlined by the green frame in the 100× image are HMX particles. Compared to the AP particles, the HMX particles show relatively better bonding with the matrix, although interfacial debonding can still be observed in some of the HMX particles. A portion containing HMX particles was selected for higher-magnification observation. The 2000× magnification image reveals that this HMX particle is closely bonded to the matrix, with no obvious debonding. In the 2000× image, highly spherical circular particles, such as those outlined in yellow, can be seen; these constitute spherical aluminum powder. The image shows that the Al powder bonds well with the matrix. There is only a very small amount of debonding between Al powder and the matrix in the 2000× magnification image. In summary, the bonding performance between the three types of solid filler particles and the NEPE propellant matrix can be ranked as follows: Al powder > HMX > AP. The larger AP particles are the most prone to dewetting.

In both the 100× and 2000× magnification images, numerous black circular pores of varying sizes can be observed. Analysis suggests that these pores primarily originate from two sources: firstly, gas pores formed by the release of gases from nitrate ester decomposition, and, secondly, structural defects generated during the tensile process. This phenomenon is consistent with the point-like defects observed in the micro-CT scan results, further confirming their structural characteristics at the mesoscopic scale.

#### 3.2.2. Analysis of SEM Images at Different Aging Times

**Figure 10:**
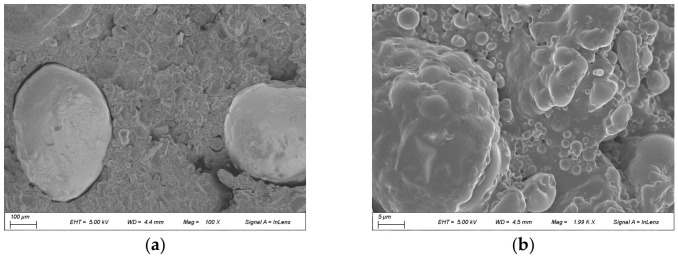
SEM scan image of the specimen aged for 1 week: (**a**) 100× and (**b**) 2000×.

For the specimens at 10 different aging timepoints, SEM scan images showing relatively significant morphological changes were selected for analysis. An SEM image of the specimen aged for 1 week is shown in Figure 10.

**Figure 11:**
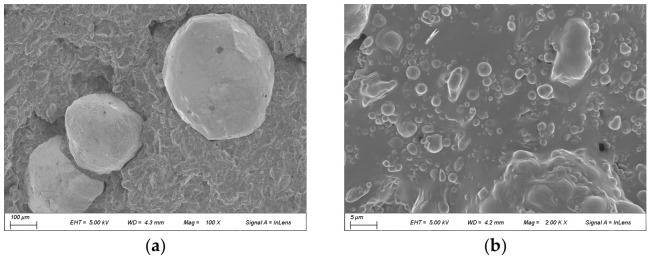
SEM image of the specimen aged for 4 weeks: (**a**) 100× and (**b**) 2000×.

Compared to the unaged specimen, the degree of dewetting in the specimen aged for 1 week was less severe. In the 100× magnification image, distinct dewetting areas between AP particles and the matrix are still visible, but the dewetting area has significantly decreased. The boundary contours of the HMX particles remain clear, but the number of particles exhibiting dewetting has greatly diminished, with most HMX particles maintaining good adhesion to the matrix. The 2000× magnification image shows that the spherical aluminum powder remains closely bonded to the matrix, with no significant dewetting observed. The NEPE propellant specimen aged for 4 weeks is shown in Figure 11.

**Figure 12:**
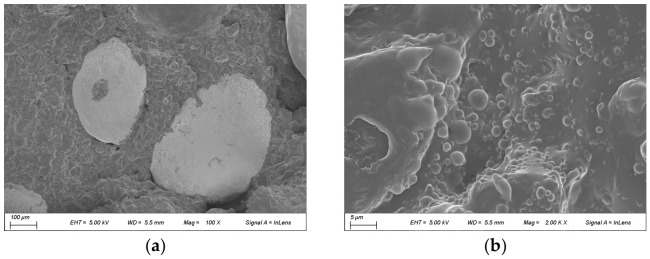
SEM image of the specimen aged for 6 weeks: (**a**) 100× and (**b**) 2000×.

The 100× magnification image shown in Figure 11 reveals that at this aging stage, the matrix enveloped the AP particles relatively tightly, with only localized minor dewetting present. HMX particles are difficult to distinguish clearly at this magnification level, indicating good adhesion with the matrix. In the 2000× magnification image, there is no significant dewetting between the matrix and the filler particles. There are significantly fewer pores relative to the 1-week-aged specimen, and the matrix exhibits a more continuous and dense morphology. The tensile fracture surface of the NEPE propellant specimen aged for 6 weeks is shown in Figure 12.

**Figure 13:**
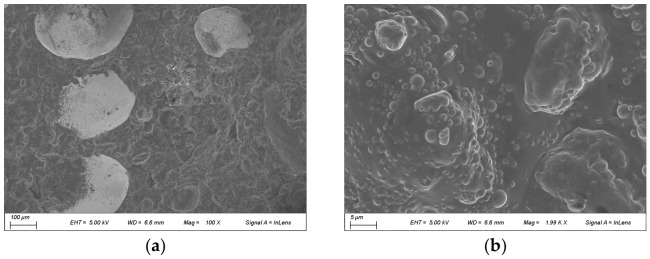
SEM image of the specimen aged for 9 weeks: (**a**) 100× and (**b**) 2000×.

In the 100× magnification image of the specimen aged for 6 weeks, the overall bonding state at the matrix/filler interface is similar to that of the 4-week-aged specimen. However, in the 6-week-aged image, a distinctly darker material, identifiable as residual binder matrix, is visible on the left AP particle. This phenomenon indicates that the interfacial bond strength at this location is greater than the inherent strength of the matrix material itself, leading to fracturing within the matrix rather than at the interface. In the 2000× magnification image, pores are essentially absent. The SEM scan image of the specimen aged for 9 weeks is shown in Figure 13.

**Figure 14:**
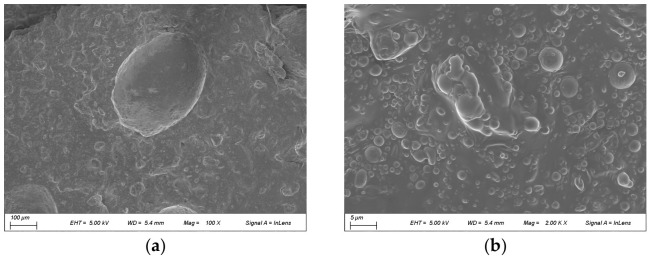
SEM image of the specimen aged for 12 weeks: (**a**) 100× and (**b**) 2000×.

As shown in Figure 13a, by the ninth week of aging, the specimen’s matrix began to appear pasty and softened, enveloping the solid filler particles more tightly. Particle contours became blurred. Compared to the specimens aged for shorter amounts of time, the matrix at this stage had significantly softened. A substantial amount of residual matrix adhered to the surface of the fractured AP particles, reflecting a decrease in the matrix’s strength. The 2000× magnification image further confirms the significant matrix softening. The SEM scan image of the fracture surface of the specimen aged for 12 weeks is shown in Figure 14.

Compared to the specimen aged for 9 weeks, the specimen aged for 12 weeks exhibited more pronounced matrix softening, showing a clear trend towards a paste-like consistency. In the 100× magnification image, a distinct AP particle is visible in the upper-middle section, but it is completely covered in black, indicating that the AP particle is now fully enveloped by the matrix. The above phenomena suggest that, at this aging stage, the location of tensile fracturing at the matrix/filler interface has shifted from the interfacial bond to the interior of the matrix, resulting in residual matrix material adhering to the surface of the solid filler particles. Relative to the specimen aged for 9 weeks, the 2000× magnification image shows no significant morphological changes.

In summary, during aging, the matrix gradually softens and becomes more viscous, and its bonding with the filler improves. Along with the test results in references [26,27], these results show that the main changes that occur during the aging of NEPE propellant are a decrease in crosslinking density and the decomposition of nitrate plasticizers. Both of these changes lead to the softening of the matrix and a reduction in its plasticity. The mesoscopic observation results in this paper are consistent with this phenomenon, indicating that after softening, the matrix is more likely to adhere to the solid filler, making it less prone to moisture removal in NEPE propellant.

## 4. Conclusions

To systematically investigate the evolution of matrix/filler interfacial adhesion and dewetting behavior during aging, micro-CT was employed to obtain three-dimensional (3D) images of NEPE propellant specimens subjected to a fixed 20% strain. Characteristic changes in the 3D pore structure and variations in porosity were analyzed. In addition, SEM was used to examine the tensile fracture surfaces of specimens aged for different durations, allowing observation of the adhesion between solid fillers (AP, HMX, and Al) and the matrix as well as the effects of aging on matrix properties and interfacial performance. The main conclusions are as follows:

1. Micro-CT images of the unaged specimens revealed prominent petal-like dewetting defects, which were large and numerous. With progressive aging, these defects gradually diminished in number, thickness, and overall volume. The calculated porosity exhibited an overall decline, decreasing rapidly within the first 0–5 weeks and then remaining relatively stable from weeks 5 to 12.
2. SEM observations of the tensile fracture surfaces showed that with an increase in aging time, the matrix progressively softened and increasingly enveloped the solid fillers, blurring particle boundaries. Dewetting areas decreased markedly during the early aging stages. By the 6th week, residual matrix adhering to filler particles became evident. After 9 weeks, the matrix appeared paste-like, with substantial matrix residue covering the fillers. At 12 weeks, AP particles were entirely coated by the matrix, indicating that interfacial strength had exceeded matrix cohesion, resulting in matrix-dominated fractures.
3. Our meso- and microscopic observations demonstrate that in NEPE propellant, with prolonged aging, dewetting becomes less pronounced, and matrix/filler interfacial adhesion improves.

## Figures and Tables

**Table 1:** Components and content of NEPE propellant.

Component	Adhesive System	Plasticizer	AP	HMX	Al Powder
content	5–8%	10–20%	10–15%	40–50%	15–20%

## Data Availability

The raw data can be provided by the corresponding author upon request.

## Abbreviations

The following abbreviations are used in this manuscript:

NEPE	Nitrate-Ester-Plasticized Polyether
CT	Computed Tomography
SEM	Scanning Electron Microscope
HMX	Cyclotetramethylenetetranitramine
AP	Ammonium Perchlorate
NG	Nitroglycerin
BTTN	1,2,4-butanetriol Trinitrate
PTFE	Polytetrafluoroethylene
